# Identifying of 22q11.2 variations in Chinese patients with development delay

**DOI:** 10.1186/s12920-020-00849-z

**Published:** 2021-01-22

**Authors:** Yuanyuan Zhang, Xiaoliang Liu, Haiming Gao, Rong He, Yanyan Zhao

**Affiliations:** Department of Clinical Genetics, Shengjing Hospital of China Medical University, Shenyang, China

**Keywords:** 22q11.2 variation, Development delay, Clinical phenotype, MLPA

## Abstract

**Background:**

22q11.2 variation is a significant genetic factor relating to development delay and/or intellectual disability. However, the prevalence, genetic characteristics and clinical phenotype in Chinese patients are unknown.

**Methods:**

In total 6034 patients with development delay and/or intellectual disability were screened by multiplex ligation-dependent probe amplification (MLPA) P245 and G-band karyotyping. The positive patients with 22q11.2 imbalance were confirmed by MLPA P250 assay.

**Results:**

52 (0.86%) patients were found to carry different levels of 22q11.2 variations, in which 37 cases (71.2%) had heterozygous deletions, whereas 15 (28.8%) had heterogeneous duplications. 34 cases (65.4%) carried typical imbalance from low copy repeat (LCR) 22 A to D. The other cases had atypical variations, relating to LCR22 A-B, LCR22 C-D, LCR22 B-D, LCR22 D-E, LCR22 E-F and LCR22 B-F region. The phenotypes of these 52 patients were variable, including development delay, language delay, facial anomalies, heart defects, psychiatric/behavior problems, epilepsy, periventricular leukomalacia, hearing impairment, growth delay etc.

**Conclusion:**

These data revealed the prevalence and variability of 22q11.2 genomic imbalance in Chinese patients with development delay and/or intellectual disability. It suggested that genetic detection of 22q11.2 is necessary, especially for the patients with mental retardation and development disorders, which deserves the attention of all pediatricians in their daily work.

## Background

Developmental delay and/or intellectual disability affects an estimated 1–3% of the population and is caused by genetic factors such as chromosomal rearrangements in the 25% of patients [1]. Chromosomal microdeletion / microduplication (< 5 Mb) explains at least 7.8% of subjects with development delay and/or intellectual disability [2]. Clinically well described syndromes, involving multiple disease genes, have been established as DiGeorge syndrome (22q11.2 microdeletion), Williams Beuren syndrome (7q11.23 microdeletion), Prader–Willi / Angelman syndromes (15q11.2 microdeletion), among others.

22q11.2 microdeletion syndrome is a genetic disorder caused by heterozygous deletions on chromosome 22q11.2 and is considered the most frequent chromosome microdeletion syndrome, with an overall prevalence of about 1: 6000 in whites, blacks and Asians [3]. The phenotypic characteristics of 22q11.2 deletion syndrome is highly variable, commonly it includes congenital heart malformations, palatal abnormalities, immune deficiency, characteristic facial features, developmental delay and learning difficulties [4–6]. Unlike the 22q11.2 deletions, duplications within this region are rarely reported. Less is known about the highly variable phenotypes linked to 22q11.2 duplication, but it appears to be associated with elevated rates of language delay and psychiatric/behavior problems [7, 8]. Much has yet to be learned regarding the reasons for similarities and widely variable features for individuals with 22q11.2 imbalance.

The MLPA P245 assay is conventionally used to screen patients presenting with developmental delay and/or intellectual disability for 31 kinds of common microdeletion or microduplication syndromes. P245 probemix includes 5 probes within 22q11.2 region, which are CLDN5, GP1BB, SNAP29, PPIL2 and RTDR1. Ratio anomalies of these probes are described as LCR22 A-B, LCR22 C-D and distal 22q11 corresponding to the known low copy repeat (LCR) regions that are involved in the imbalance. The confirmation of 22q11.2 variation is mostly done with the standard MLPA P250 assay which has 24 dense probes located in this region and can give more reproducible results and more detailed information about the size of the deletion or duplication.

This study presents an overview of the results obtained from use of MLPA P245 and P250 assays for diagnosis on patients with 22q11.2 imbalance. The aim of this study was to estimate the prevalence and detailed genetic characterization of 22q11.2 variations in Chinese patients with development delay and/or intellectual disability. We also aimed to explore possible genotype–phenotype relationship in order to provide basic information for clinical evaluation.

## Methods

### Patients and samples

All the 6034 cases enrolled in this study were recruited between July 2013 and December 2019 through the outpatients of developmental pediatrics and neurology pediatrics of Shengjing hospital, China Medical University. Most of these cases presented with mental retardation and/or development delay. Some specific cases were requested to test by their parents for physical examination. The whole peripheral blood were retrieved from clinical genetics of Shengjing hospital. This study was approved by Ethics Committee of Shengjing Hospital of China Medical University. Written informed consent to participate was obtained from all of the participants in this study (written informed consent to participate of individuals younger than the age of 16 was obtained from their parents or legal guardians).

### DNA extraction

Genomic DNA was extracted from the whole peripheral blood using Automatic nucleic acid extractor (Allsheng Auto-Pure 32A) with UPure Blood DNA Extraction Kit (M2002-A32) (BioBase Technologies Co., LTD) following the manufacturer’s instructions. Then the concentration was detected using a spectrophotometry method (NanoDrop 1000, Thermo Scientifific, USA), and a concentration of 10–50 ng*/μ*L DNA was prepared for following tests.

### MLPA assay

MLPA is a semi-quantitative technique that is designed to detect gene dosage variation based on multiplex PCR method. The SALSA MLPA KIT P245 and P250 (MRC Holland, Amsterdam, Netherlands) were used for MLPA analysis according to the manufacturer’s instructions. PCR amplification products were separated by capillary electrophoresis using ABI 3730 Genetic Analyser (Applied Biosystems, USA). Coffalyser. Net software (MLPA Holland, Amsterdam, Netherlands) was used to analyze and give an interpretation of the raw MLPA data. The detailed information of the probes included in MLPA kits is described in the manufacture’s instructions.

### Chromosome karyotype analysis

Conventional chromosome G-band karyotyping analysis was performed on peripheral blood in accordance with the standard procedures used for evaluating numerical and structural chromosome aberrations in the cultures of blood cells. Leica CytoVision (Leica, USA) was used to capture images of mitotic metaphase chromosomes. The karyotyping results were identified and described upon agreement of the two examiners, with reference to the International System for Human Cytogenetic Nomenclature (ISCN 2016) [9].

## Results

### Molecular analysis

**Fig. 1:**
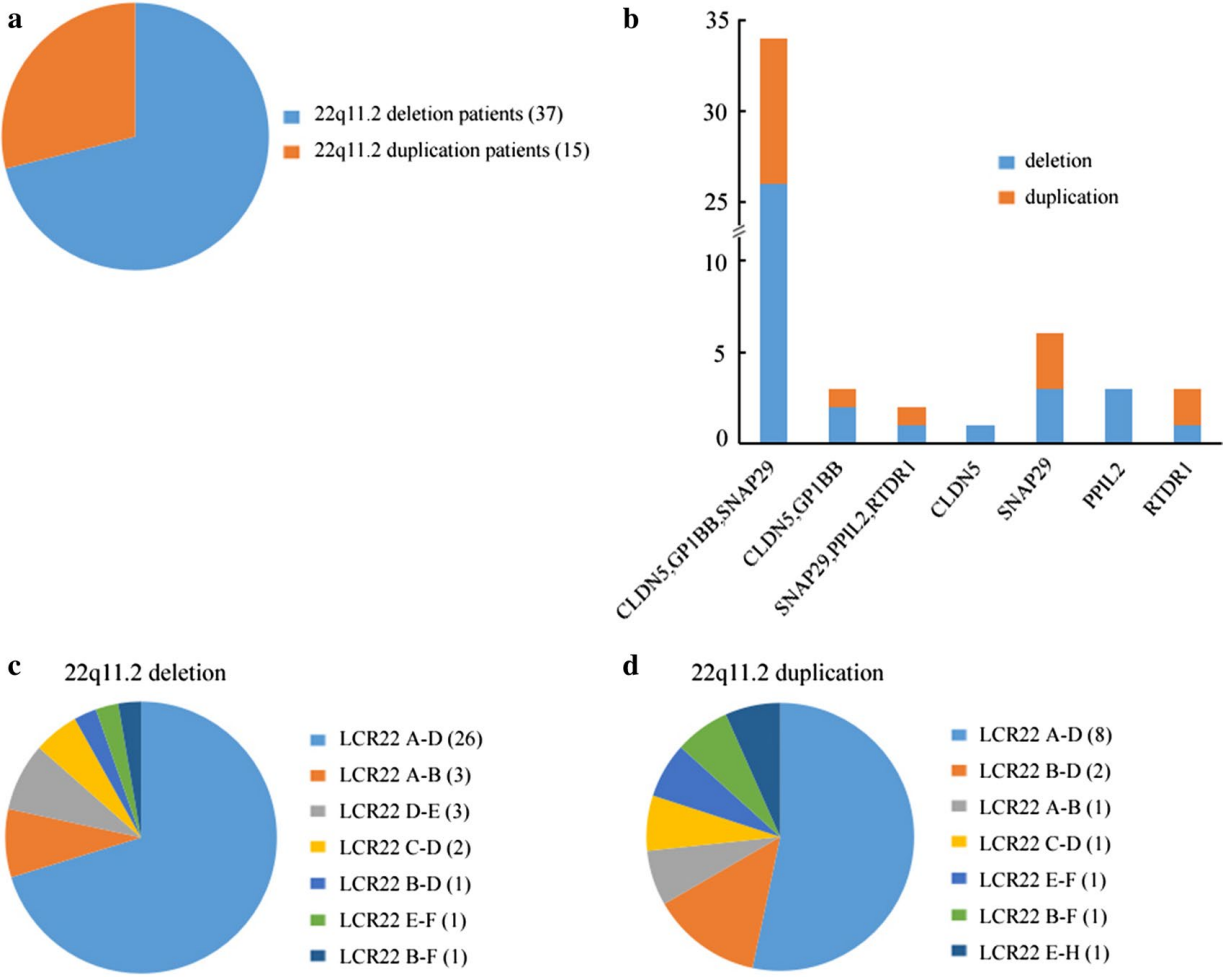
**a** The frequency of deletion and duplication in patients with 22q11.2 imbalance undergoing multiplex ligation-dependent probe amplification (MLPA) P245 analysis. **b** The frequency of abnormal probes detected by P245 assay. Y-axis represents number of patients. **c** The frequency of different levels of 22q11.2 deletions. **d** The frequency of different levels of 22q11.2 duplications. LCR, low copy repeats

Of all the 6034 cases screened by MLPA P245 assay, 52 (0.86%) were found to carry variations on chromosome 22q11.2. Their ages ranged from 11 days to 27 years. Among the 52 positive samples, 37 (71.2%) had heterozygous deletions, while the remainder (28.8%) had heterogeneous duplications (Fig. 1a). In addition, we found that (1) 34 cases (65.4%) had imbalance at CLDN5, GP1BB and SNAP29 probes including 26 cases of deletions and 8 cases of duplications; (2) 3 cases (5.8%) had imbalance at CLDN5 and GP1BB probes, of which 2 had deletions and one had duplication; (3) 2 cases (3.8%) had imbalance at SNAP29, PPIL2 and RTDR1 probes, and they had deletion and duplication, respectively. Finally, the remainder 13 cases (26.4%) had anomalies at single probe. Among them, 8 individuals had deletions involving CLDN5 (1), SNAP29 (3), PPIL2 (3) and RTDR1 (1) probes, and the other 5 individuals had duplications relating to SNAP29 (3) and RTDR1(2) probes. Details of the P245 results were showed in Fig. 1b.

**Fig. 2:**
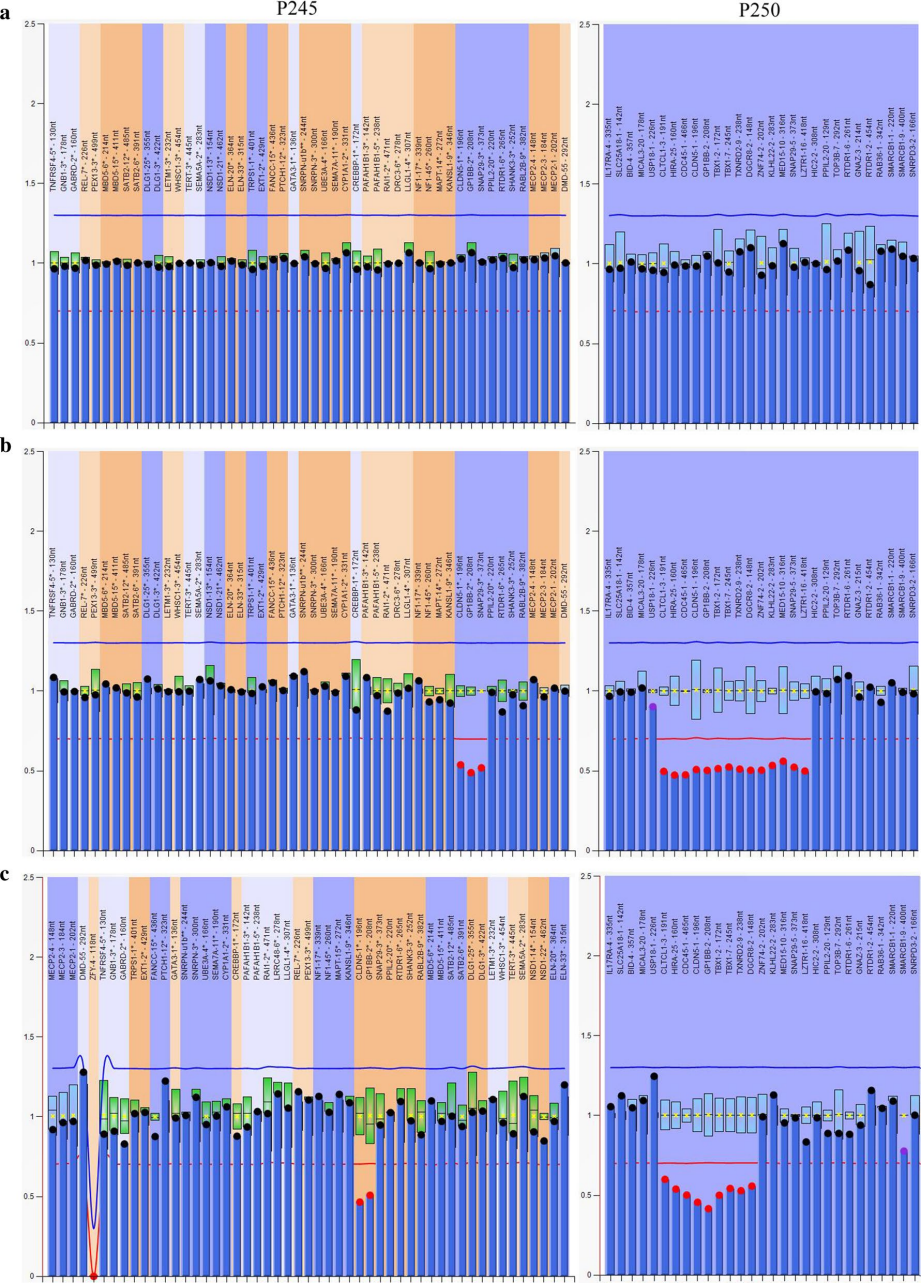

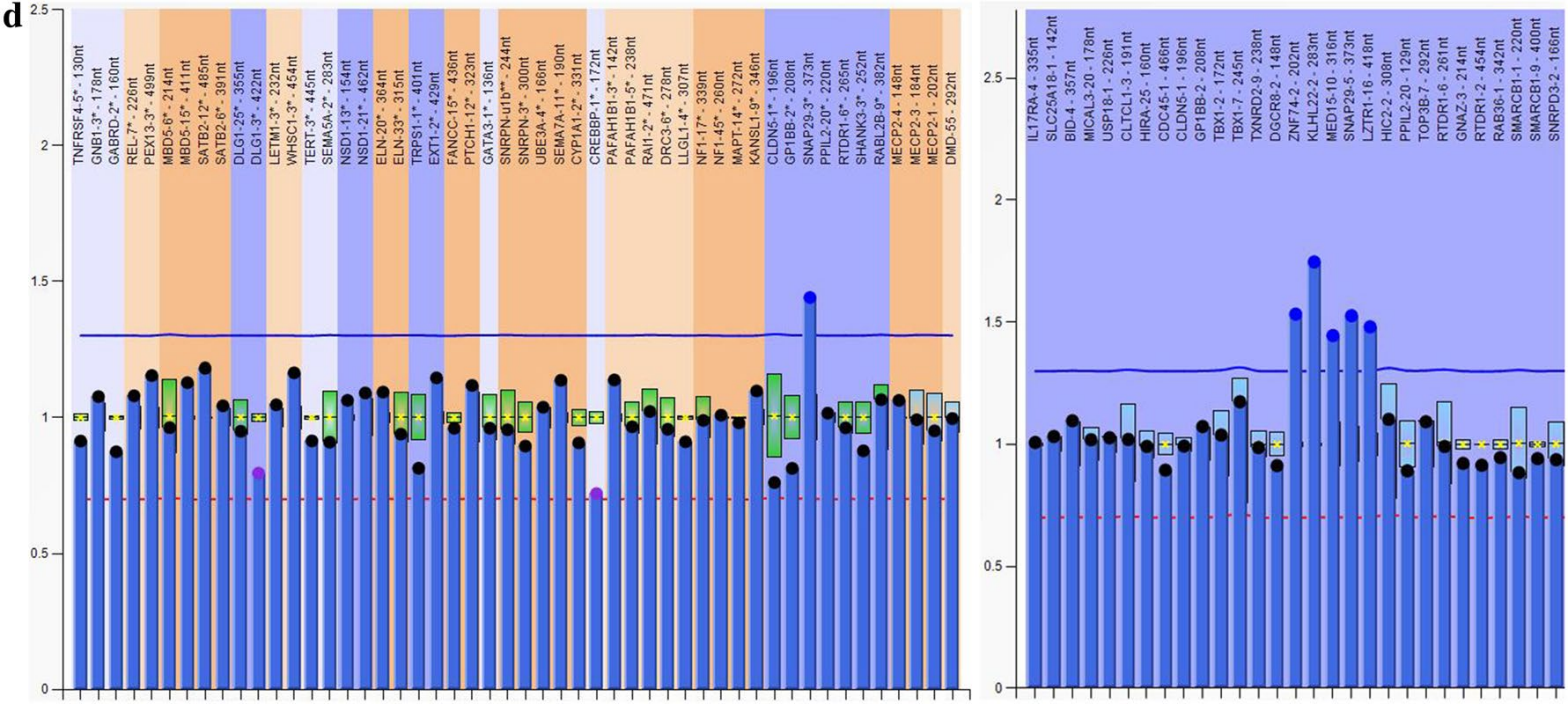
Graphs represent results analyzed by Multiplex ligation-dependent probe amplification (MLPA) in three cases and one normal control. X-axis represents MLPA probes (genes) and y-axis represents probe dosage quotient. The blue line indicates probe dosage quotient of 1.35 and any probes above this line represent duplication. The red line indicates probe dosage quotient of 0.65 and any probes below this line represent deletion. The probes between 0.85 and 1.15 are considered as normal controls. **a** A control with normal copy probes. **b** A patient carries typical deletion from CLTCL1 to LZTR1 (LCR22 A-D). LCR, low copy repeats. **c** A patient carries deletion from CLTCL1 to DGCR8 (LCR22 A-B). **d** A patient carries atypical smaller duplication from ZNF74 to LZTR1 (LCR22 B-D)

MLPA P250 assay was further used to confirm the P245 results and evaluate the imbalance region. The results indicated that all the 34 cases with imbalance at CLDN5, GP1BB and SNAP29 probes carried typical deletions or duplications from CLTCL1 to LZTR1 (LCR22 A-D). The 3 cases with imbalance at CLDN5 and GP1BB probes carried anomalies from CLTCL1 to DGCR8 (LCR22 A-B) and that 2 cases with imbalance at SNAP29, PPIL2 and RTDR1 probes carried variations from ZNF74 to RAB36 (LCR22 B-F). The other 13 cases with single probe abnormal were confirmed to carry atypical smaller variations, including LCR22 A-B, LCR22 C-D, LCR22 B-D, LCR22 D-E and LCR22 E–F region. Details were described in Fig. 1c, d. Additional probes outside of chromosome 22q11.2 contained in P250 kit (4q34, 8p23, 9q34.3, 10p15, 17p13.3 and 22q13) did not reveal additional copy number variations (CNVs). Figure 2 showed representative data from three patients and one normal control analyzed by MLPA.

To make clear if these 52 patients were inherited, their parents were analyzed by p245 assay as well. We found 4 cases were inherited from an affected parent while 30 cases were de novo. The other 18 cases were unknown due to the DNA samples from their parents were unavailable (Additional file 1: Table S1).

### Karyotype analysis

Based on the results of conventional chromosome G-band karyotyping analysis, we found 51 of 52 samples with 22q11.2 imbalance showed normal karyotypes as 46,XX or 46,XY. The remaining child who had duplication from LCR22 E to H showed 47,XY, + 21 (Additional file 1: Table S1). It proved again that the 22q11.2 deletion or duplication cannot be identified by routine analysis of G-banded chromosomes or other conventional cytogenetic banding techniques.

### Genotype–Phenotype analysis

For those 26 cases with typical LCR22 A-D deletions, the major phenotypes were development delay (10/26, 38.5%), language delay (9/26, 34.6%), craniofacial features (8/26, 30.8%) and heart defects (6/26, 23.1%). Craniofacial features contained ear deformities (3), asymmetric crying facies (2), mouth deformities (1), cleft palate (1) and microcephaly (1). Heart defects included ventricular septal defect (3), patent ductus arteriosus (2) and foramen ovale opening (1). Some patients also showed other manifestations such as psychiatric/behavior problems (3), epilepsy (3), periventricular leukomalacia (3), intellectual disability (2), hearing impairment (2), growth delay (2), gastroesophageal reflux (1), extracerebral space widen (1) and limb pain (1). Two of the 19 male patients were diagnosed as cryptorchidism. In particular, 3 cases did not have any syndrome at the current stage. Two of them were chrildren, and they were requested by their parents for physical examination. The other one was a 27 years old woman, she wasn't identified until she came to outpatient for the reason that she conceived 3 times while the fetus had tetralogy of fallot, hydronephrosis and ventricular septal defect, respectively.

The 8 cases with LCR22 A-D duplications presented similar spectrum of clinical symptoms with deletions, such as language delay (3), development retardation (2), epilepsy (2), periventricular leukomalacia (2), heart defect with pulmonary hypertension (1), intellectual disability (1), hearing impairment (1), gastroesophageal reflux (1) and extracerebral space widen (1). Some cases also had other manifestations containing corpus callosum thin (1), hiatal hernia (1), cerebellar tonsil hernia (1), hypoeonia (1), involuntary movement (1).

In those cases with atypical smaller variations, language delay (55.6%, 10/18) is the most common manifestation, following by development delay (33.3%, 6/18). Particularly, the 3 cases with LCR22 A-B deletion all had language delay. Specific cases also had other associated phenotypes like growth delay, psychiatric/behavior problems, epilepsy, intellectual disability, ect. The 3 cases who had duplications in LCR22 C-D or B-D presented with language delay, psychiatric/behavior problems and even autism. In Table 1 is shown the detailed clinical data of phenotypes of the patients with genetic variations within the 22q11.2 region.

**Table 1:** Phenotypic characteristics of 52 cases with 22q11.2 variations

Phenotypic characteristics	22q11.2 Deletions (n = 37)							22q11.2 Duplications (n = 15)						
	A-D (n/26)	A-B (n/3)	D-E (n/3)	C-D (n/2)	B-D (n/1)	E–F (n/1)	B-F (n/1)	A-D (n/8)	A-B (n/1)	C-D (n/1)	B-D (n/2)	E–F (n/1)	B-F (n/1)	E–H (n/1)
Development delay	10		1		1	1	1	2				1	1	
Language delay	9	3	1	1			1	3			2	1	1	
Craniofacial anomalies	8	1						1						1
Heart defect	6	1						1						1
Psychiatric/Behavior problems	3										2			
Epilepsy	3	1		1				2						
Periventricular leukomalacia	3							2						
Growth delay	2		1		1		1							
Intellectual disability	2		1	1				1						
Hearing impairment	2							1					1	
Cryptorchidism	2													
Gastroesophageal reflux	1							1						1
Extracerebral space widen	1							1						
Limb pain	1													
Autism										1				
Corpus callosum thin								1						
Hiatus hernia								1						
Cerebellar tonsil hernia								1						
Hypotonia								1						
Pulmonary hypertension								1						
Involuntary movement								1						
Headache									1					
Congenital cataract					1									
Hyperbilirubinemia														1

## Discussion

Patients with 22q11.2 imbalance has broad prototypical variability, from no abnormalities to severe mental retardation with multiple congenital malformations. Because of the strong variance in phenotype, some patients with a deletion or duplication are not immediately identified or diagnosed at birth. In our study, MLPA P245 assay was applied to screen in the 6034 cases who presented with development retardation with or without other disorders. The results indicated that 22q11.2 is the third most frequent pathogenic CNVs with a frequency of 0.86%, following 15q11.2 (1.36%) and 7q11.23 (1.18%). The prevalence of 22q11.2 deletions and duplications were 0.61% (37/6034) and 0.25% (15/6034), respectively, which were similar to the data reported in the literature (0.69 and 0.36%) [10].

The high frequency of 22q11.2 copy number changes is attributed to the presence of a cluster of LCRs in 22q11.2 [11, 12]. There are eight LCR blocks named as LCR22 A to LCR22 H in the 22q11.2 region [12]. Approximately 85–90% of individuals with 22q11.2 deletion syndrome have a 3 Mb deletion spanning from LCR22 A to D, while 8–10% have a nested 1.5 Mb deletion extending only from LCR22 A to B [13, 14]. In our study, among the 37 cases with 22q11.2 deletions, 26 (70.3%) cases carried LCR22 A-D deletions and 3 (10.8%) cases had LCR22 A-B deletions. We also identified 5 types of atypical deletions including LCR22 D-E (3/37, 8.1%), LCR22 C-D (2/37, 5.4%), LCR22 B-D (1/37, 2.7%), LCR22 E–F (1/37, 2.7%) and LCR22 B-F (1/37, 2.7%). The types of 22q11.2 deletions were more variable in our cohort and the prevalence varied largely, probably due to different sample size as well as our subjects selected as individuals with obviously developmental delay and/or mental retardation rather than congenital heart defects or craniofacial abnormality.

Studying the genotype and phenotype relationship of 22q11.2 deletion is difficult as the number of possible clinical symptoms is large and even individuals within families with the same type of deletion differ in clinical manifestation. Our subjects with LCR22 A-D deletion mostly presented with development delay, language delay, facial anomalies, heart defects, psychiatric/behavior problems, epilepsy and periventricular leukomalacia. Some of them also had hearing impairment, cryptorchidism etc. However, there was a 4 months old girl and a 7 years old boy who were requested by their parents for physical examination. Probably they had so mild symptoms that we couldn't identified, or they were too young to evaluate the associated phenotypes. They presented with no symptom at this stage. The underlying mechanisms of variable penetrance are not well understood at present. Many factors like genetic background, modifier genes, epigenetic changes and environmental factors may have important roles. More attention should be focused on the follow-up studies as the disorders like intellectual disability, psychiatric or behavioral problems may become obvious when they are older. Another case was a 27 years old woman, who didn't have clinical manifestation of her own. She could be classified as an asymptomatic carrier. Because of clinical variability and / or reduced penetrance, she was not identified until her three fetus were found to have congenital heart disease or hydronephrosis. As each child of an individual with 22q11.2 deletion syndrome has a 50% chance of inheriting the 22q11.2 imbalance. Therefore, genetic counseling for a pregnancy at increased risk, prenatal testing and preimplantation genetic testing are significant.

The symptoms of atypical deletions in our study mainly were language delay, development delay, intellectual disability, and growth delay. Because of the overlapping features of individuals with various 22q11.21 CNVs, the genotype–phenotype correlations could not be accurately predicted. Other factors that may impact the phenotypic similarity and variability remain to be determined. It has been reported that dysregulation of genes by loss of long-range regulatory sequences could affect either common genes and/or common developmental pathways [15, 16]. For example, long-range chromatin interaction of COMT in the proximal 22q11.2 region with genes on other chromosomes, as well as with genes in the distal 22q11.2 region may mediate similarities between typical, atypical, and distal 22q11 deletion phenotypes [15].

Of the 15 patients with 22q11.2 duplications, 8 had LCR22 A-D duplications, and the remaining 7 patients had LCR22 A-B (1/15, 6.7%), LCR22 C-D (1/15, 6.7%), LCR22 B-D (2/15, 13.3%), LCR22 E–F (1/15, 6.7%), LCR22 B-F (1/15, 6.7%) and LCR22 E–H (1/15, 6.7%), respectively. The types of duplications were also variable and the spectrum of symptoms was similar with 22q11.2 deletion syndrome. Our cases with LCR22 A-D duplications mainly presented with language delay, development delay, epilepsy, and periventricular leukomalacia. Intriguingly, we noticed that the 3 patients with only SNAP29 probe duplication in P245 assay, who were confirmed to have nested atypical duplications from C-D or B-D in P250 assay, presented with language delay, difficulty with social interactions and even autism. While the 3 patients with corresponding site deletion mainly showed development delay. A recent study concluded that the prevalence of neuropsychiatric disorders was higher in duplication carriers compared with deletion carriers [17]. The atypical nested LCR22 B-D duplications are associated with an increased risk for neurodevelopmental phenotypes particularly autism spectrum disorder (ASD) [18]; suggesting the critical genes related with these neurodevelopmental phenotypes including ASD may be located between LCR22 B and LCR22 D. We performed NGS on the patient with LCR22 C-D duplication, who was clinically diagnosed with autism and found a 0.36 Mb duplication containing *SNAP29* and *SERPIND1* genes. SERPIND1 is very weakly expressed in brain tissues. Whereas SNAP29 has been found to negatively modulate neurotransmitter release and contributes to schizophrenia and autism spectrum disorder [19]. It encodes a soluble NSF-attachment protein (SNAP) receptor (i.e., SNARE) protein that competes with a-SNAP for binding to SNARE complexes, thus reducing SNARE protein recycling and synaptic vesicle availability [20]. Mutations in SNAP29 result in variable expressivity and incomplete penetrance. Patients with homozygous mutations in SNAP29 are responsible for CEDNIK (cerebral dysgenesis, neuropathy, ichthyosis, and keratoderma) syndrome [21], which has a number of clinical manifestations, some of which overlap with those found in 22q11.2 deletion syndrome. SNAP29 was reported to be abnormal in 90% of patients with 22q11.2 deletion syndrome. In our cohort, *SNAP29* gene exhibited the highest frequency of variation among the 37 deletion and 15 duplication cases, with the incidence were 81.1% and 80%, respectively. Additional functional studies are necessary, to evaluate the role of SNAP29 in autism and the consequence of other genes expressed in LCR22 B-D in neurodevelopmental phenotypes.

In contrast to the prevalence of language delay, developmental retardation, cognitive impairment and behavioral problems in patients with LCR22 A-D and A-B imbalance, we found no incidence of cardiac defects and facial anomalies in any of our symptomatic patients with other atypical types of variation. We therefore postulated that the responsible genes for such factors are likely to lie within LCR22A to LCR22B region. Of note, *HIRA*, *TBX1* and *DGCR8* are considered candidate critical genes for major phenotypes associated with 22q11.2 disorders, especially the cardiac defects [22–26]. In addition, due to the fact that most of our patients were children, some clinical manifestations could be highlighted in the future such as phychiatric / behaviour problems, autism, headache, learning difficulties etc. So reassessment is required in these children in future follow up.

## Conclusion

Taken together, the present study revealed the exact prevalence of 22q11.2 variations in children with development delay and/or intellectual disability in north of China. We found variable disorders within the 22q11.2 region including typical and atypical variations. The atypical variations including smaller deletions/duplications and rare deletions/duplications found may be linked to various phenotypes of development delay and related clinical manifestations. Early and accurate detection of 22q11.2 deletion and duplication syndromes in patients is important especially for those with mental retardation and development delay but without characteristic clinical symptoms such as cardiac defects, craniofacial abnormality, hypo- or aplasia of the thymus and hypoparathyroidism. These important findings deserve the attention of all pediatricians in their daily work.

## Supplementary Information

**Additional file 1.** Detailed clinical data and genetic results of 52 cases with 22q11.2 variations.

## Data Availability

Data derived from MLPA assay were analyzed and interpreted by Coffalyser. Net, which is available at (https://www.mrcholland.com/technology/software/coffalyser-net). Detailed clinical data and genetic results of all cases are provided in Additional file 1: Table S1. The hg19 human reference genomic sequence dataset used in our study was from USCS Genome Brower repository (http://genome.ucsc.edu/cgi-bin/hgTracks?db=hg19&lastVirtModeType=default&lastVirtModeExtraState=&virtModeType=default&virtMode=0&nonVirtPosition=&position=chr22%3A18752704%2D25239487&hgsid=962156695_kliTac78I6Qs5H1K70fRaCAMOAiM). Raw data of MLPA assay obtained in our study is available from the corresponding author on request.

## Abbreviations

ASD: Autism spectrum disorder
CEDNIK syndrom: Cerebral dysgenesis, neuropathy, ichthyosis, and keratoderma syndrome
CLDN5: Claudin 5
CLTCL1: Clathrin heavy chain like 1
CNV: Copy number variation
COMT: Catechol-O-methyltransferase
DGCR8: DGCR8 microprocessor complex subunit
GP1BB: Glycoprotein Ib platelet subunit beta
HIRA: Histone cell cycle regulator
LCR: Low copy repeat
LZTR1: Leucine zipper like transcription regulator 1
MAPK1: Mitogen-activated protein kinase 1
MLPA: Multiplex ligation-dependent probe amplification
NSF: *N*-Ethylmaleimide-sensitive factor
PPIL2: Peptidylprolyl isomerase like 2
RAB36: RAB36, member RAS oncogene family
RTDR1 (RSPH14): Radial spoke head 14 homolog
SERPIND1: Serpin family D member 1
SNAP: Soluble NSF attachment protein
SNAP29: Synaptosome associated protein 29
SNARE: SNAP receptor
TBX1: T-box transcription factor 1
ZNF74: Zinc finger protein 74
