# Supplementary material for: Identifying of 22q11.2 variations in Chinese patients with development delay
Source: BMC Med Genomics. 2021 Jan 22;14:26. doi: 10.1186/s12920-020-00849-z (PMC7821542; doi:10.1186/s12920-020-00849-z)
Supplement: Supplementary file 1 — Additional file 1. Detailed clinical data and genetic results of 52 cases with 22q11.2 variations. [file 12920_2020_849_MOESM1_ESM.docx]

Supplementary Table 1. Detailed clinical data and genetic results of 52 cases with 22q11.2 variations.

| **Patient No.** | **Gender/Age** | **Deletions or Duplications** | **P245 assay** | **P250 assay** | **Karyotype** | **Phenotypes** | **Inheritance** |
| --- | --- | --- | --- | --- | --- | --- | --- |
| 1 | F/11d | deletions | CLDN5, GP1BB2, SNAP29 | LCR22 A-D (CLTCL1-LZTR1) | 46,XX | PDA, periventricular leukomalacia | unknown |
| 2 | M/17d | deletions | CLDN5, GP1BB2, SNAP29 | LCR22 A-D (CLTCL1-LZTR1) | 46,XY | periventricular leukomalacia, gastroesophageal reflux, PFO | de novo |
| 3 | M/9y | deletions | CLDN5, GP1BB2, SNAP29 | LCR22 A-D (CLTCL1-LZTR1) | 46,XY | epilepsy | de novo |
| 4 | F/12y | deletions | CLDN5, GP1BB2, SNAP29 | LCR22 A-D (CLTCL1-LZTR1) | 46,XX | VSD, periventricular leukomalacia | de novo |
| 5 | F/27y | deletions | CLDN5, GP1BB2, SNAP29 | LCR22 A-D (CLTCL1-LZTR1) | 46,XX | none | unknown |
| 6 | M/2y | deletions | CLDN5, GP1BB2, SNAP29 | LCR22 A-D (CLTCL1-LZTR1) | 46,XY | language delay, psychiatric/behavior problems, microtia | unknown |
| 7 | M/13m | deletions | CLDN5, GP1BB2, SNAP29 | LCR22 A-D (CLTCL1-LZTR1) | 46,XY | hearing impairment, development delay, language delay | unknown |
| 8 | M/7y | deletions | CLDN5, GP1BB2, SNAP29 | LCR22 A-D (CLTCL1-LZTR1) | 46,XY | none | de novo |
| 9 | M/2y | deletions | CLDN5, GP1BB2, SNAP29 | LCR22 A-D (CLTCL1-LZTR1) | 46,XY | VSD, language delay, low auricular position, | de novo |
| 10 | M/2m | deletions | CLDN5, GP1BB2, SNAP29 | LCR22 A-D (CLTCL1-LZTR1) | 46,XY | development delay, extracerebral space widen, asymmetric crying face, unilateral large ear,hearing impairment | inherited |
| 11 | M/17m | deletions | CLDN5, GP1BB2, SNAP29 | LCR22 A-D (CLTCL1-LZTR1) | 46,XY | asymmetric crying face | de novo |
| 12 | M/10m | deletions | CLDN5, GP1BB2, SNAP29 | LCR22 A-D (CLTCL1-LZTR1) | 46,XY | development delay | de novo |
| 13 | M/7y | deletions | CLDN5, GP1BB2, SNAP29 | LCR22 A-D (CLTCL1-LZTR1) | 46,XY | epilepsy, language delay, VSD | unknown |
| 14 | F/16m | deletions | CLDN5, GP1BB2, SNAP29 | LCR22 A-D (CLTCL1-LZTR1) | 46,XX | development delay; limb pain, microcephalia, psychiatric/behavior problems | de novo |
| 15 | F/4y | deletions | CLDN5, GP1BB2, SNAP29 | LCR22 A-D (CLTCL1-LZTR1) | 46,XX | language delay | de novo |
| 16 | M/3y | deletions | CLDN5, GP1BB2, SNAP29 | LCR22 A-D (CLTCL1-LZTR1) | 46,XY | language delay | de novo |
| 17 | M/17m | deletions | CLDN5, GP1BB2, SNAP29 | LCR22 A-D (CLTCL1-LZTR1) | 46,XY | development delay | unknown |
| 18 | F/4m | deletions | CLDN5, GP1BB2, SNAP29 | LCR22 A-D (CLTCL1-LZTR1) | 46,XX | none | unknown |
| 19 | M/10y | deletions | CLDN5, GP1BB2, SNAP29 | LCR22 A-D (CLTCL1-LZTR1) | 46,XY | short stature, development delay, study difficult | de novo |
| 20 | M/9y | deletions | CLDN5, GP1BB2, SNAP29 | LCR22 A-D (CLTCL1-LZTR1) | 46,XY | cleft palate, development delay, study difficult, language delay | de novo |
| 21 | M/2y | deletions | CLDN5, GP1BB2, SNAP29 | LCR22 A-D (CLTCL1-LZTR1) | 46,XY | language delay, psychiatric/behavior problems | unknown |
| 22 | M/8m | deletions | CLDN5, GP1BB2, SNAP29 | LCR22 A-D (CLTCL1-LZTR1) | 46,XY | development delay, cryptorchidism, growth delay | de novo |
| 23 | F/6y | deletions | CLDN5, GP1BB2, SNAP29 | LCR22 A-D (CLTCL1-LZTR1) | 46,XX | development delay, language delay | de novo |
| 24 | M/6y | deletions | CLDN5, GP1BB2, SNAP29 | LCR22 A-D (CLTCL1-LZTR1) | 46,XY | language delay, PDA | de novo |
| 25 | F/12d | deletions | CLDN5, GP1BB2, SNAP29 | LCR22 A-D (CLTCL1-LZTR1) | 46,XX | epilepsy | de novo |
| 26 | M/15m | deletions | CLDN5, GP1BB2, SNAP29 | LCR22 A-D (CLTCL1-LZTR1) | 46,XY | cryptorchidism, fish-mouth | de novo |
| 27 | M/3y | duplications | CLDN5, GP1BB2, SNAP29 | LCR22 A-D (CLTCL1-LZTR1) | 46,XY | epilepsy, periventricular leukomalacia | unknown |
| 28 | M/3y | duplications | CLDN5, GP1BB2, SNAP29 | LCR22 A-D (CLTCL1-LZTR1) | 46,XY | periventricular leukomalacia, corpus callosum thin, language delay, intelligence disability, esostasis | unknown |
| 29 | F/3m | duplications | CLDN5, GP1BB2, SNAP29 | LCR22 A-D (CLTCL1-LZTR1) | 46,XX | gastroesophageal reflux, hiatus hernia, VSD, PFO, PH, extracerebral space widen | de novo |
| 30 | M/13y | duplications | CLDN5, GP1BB2, SNAP29 | LCR22 A-D (CLTCL1-LZTR1) | 46,XY | involuntary moveme | inherited |
| 31 | M/17m | duplications | CLDN5, GP1BB2, SNAP29 | LCR22 A-D (CLTCL1-LZTR1) | 46,XY | language delay, development delay | inherited |
| 32 | M/11y | duplications | CLDN5, GP1BB2, SNAP29 | LCR22 A-D (CLTCL1-LZTR1) | 46,XY | epilepsy | unknown |
| 33 | M/9m | duplications | CLDN5, GP1BB2, SNAP29 | LCR22 A-D (CLTCL1-LZTR1) | 46,XY | hypotonia | de novo |
| 34 | M/2y | duplications | CLDN5, GP1BB2, SNAP29 | LCR22 A-D (CLTCL1-LZTR1) | 46,XY | development delay, language delay, cerebellar tonsil hernia, hearing impairment | de novo |
| 35 | F/2y | deletions | CLDN5, GP1BB2 | LCR22 A-B (CLTCL1-DGCR8) | 46,XX | epilepsy, language delay | de novo |
| 36 | M/2y | deletions | CLDN5,GP1BB2 | LCR22 A-B (CLTCL1-DGCR8) | 46,XY | language delay, VSD. facial anomalies | de novo |
| 37 | F/6y | duplications | CLDN5, GP1BB2 | LCR22 A-B (CLTCL1-DGCR8) | 46,XX | headache | inherited |
| 38 | F/3y | deletions | SNAP29, PPIL2, RTDR1 | LCR22 B-F (ZNF74-RAB36) | 46,XX | development dealy, language delay, growth delay | de novo |
| 39 | F/13m | duplications | SNAP29, PPIL2, RTDR1 | LCR22 B-F (ZNF74-RAB36) | 46,XX | development delay, language delay, hearing impairment | unknown |
| 40 | M/2y | deletions | CLDN5 | LCR22 A-B (CLTCL1-CLDN5) | 46,XY | language delay | unknown |
| 41 | F/9y | deletions | SNAP29 | LCR22 C-D (SNAP29-LZTR1) | 46,XX | epilepsy, intellectual disability | unknown |
| 42 | M/2y | deletions | SNAP29 | LCR22 C-D (SNAP29) | 46,XY | language delay | unknown |
| 43 | M/7m | deletions | SNAP29 | LCR22 B-D (ZNF74-LZTR1) | 46,XY | development delay, congenital cataract, growth delay | de novo |
| 44 | M/7y | duplications | SNAP29 | LCR22 C-D (SNAP29) | 46,XY | autism | de novo |
| 45 | F/3y | duplications | SNAP29 | LCR B-D (ZNF74-LZTR1) | 46,XX | language delay, psychiatric/behavior problems | de novo |
| 46 | M/3y | duplications | SNAP29 | LCR22 B-D (ZNF74-SNAP29) | 46,XY | language delay, psychiatric/behavior problems | unknown |
| 47 | M/2y | deletions | PPIL2 | LCR22 D-E (HIC2-PPIL2) | 46,XY | development dealy | unknown |
| 48 | F/4y | deletions | PPIL2 | LCR22 D-E (HIC2-TOP3B) | 46,XX | development delay, intellectual disability | unknown |
| 49 | F/12m | deletions | PPIL2 | LCR22 D-E (HIC2-TOP3B) | 46,XX | growth delay | de novo |
| 50 | F/12m | deletions | RTDR1 | LCR22 E-F (RTDR1-RAB36) | 46,XX | development delay | de novo |
| 51 | F/18m | duplications | RTDR1 | LCR22 E-F (RTDR1-RAB36) | 46,XX | development delay, language delay | de novo |
| 52 | M/2m | duplications | RTDR1 | LCR22 E-H (RTDR1-SNRPD) | 47,XY,+21 | facial anomalies, Down's syndrome, PDA, gastroesophageal reflux, hypotonia, hyperbilirubinemia | de novo |

PDA: Patent Ductus Arteriosus, PFO: patent foramen ovale, VSD: ventricular septal defect, PH: pulmonary hypertension
